# Prospects of molecular hydrogen in cancer prevention and treatment

**DOI:** 10.1007/s00432-024-05685-7

**Published:** 2024-03-31

**Authors:** Wenchang Zhou, Jie Zhang, Wankun Chen, Changhong Miao

**Affiliations:** 1 Department of Anesthesiology; Cancer Center, Zhongshan Hospital, Fudan University, No. 180 Feng-Lin Road, Shanghai, 200032 China; 2 Shanghai Key Laboratory of Perioperative Stress and Protection, Shanghai, China

**Keywords:** Cancer treatment, Hydrogen, Oxidative stress, Tumor immunity, Intestinal flora

## Abstract

Gas signaling molecules, including carbon monoxide (CO), nitric oxide (NO), and hydrogen sulfide (H_2_S), have been shown to have cancer therapeutic potential, pointing to a new direction for cancer treatment. In recent years, a series of studies have confirmed that hydrogen (H_2_), a weakly reductive gas, also has therapeutic effects on various cancers and can mitigate oxidative stress caused by radiation and chemotherapy, reducing tissue damage and immunosuppression to improve prognosis. Meanwhile, H_2_ also has immunomodulatory effects, inhibiting T cell exhaustion and enhancing T cell anti-tumor function. It is worth noting that human intestinal flora can produce large amounts of H_2_ daily, which becomes a natural barrier to maintaining the body’s resistance to diseases such as tumors. Although the potential anti-tumor mechanisms of H_2_ are still to be investigated, previous studies have shown that H_2_ can selectively scavenge highly toxic reactive oxygen species (ROS) and inhibit various ROS-dependent signaling pathways in cancer cells, thus inhibiting cancer cell proliferation and metastasis. The ROS scavenging ability of H_2_ may also be the underlying mechanism of its immunomodulatory function. In this paper, we review the significance of H_2_ produced by intestinal flora on the immune homeostasis of the body, the role of H_2_ in cancer therapy and the underlying mechanisms, and the specific application of H_2_ to provide new ideas for the comprehensive treatment of cancer patients.

## Introduction

According to recent World Health Organization (WHO) statistics (Sung et al. 2021; Wen et al. 2021; Siegel et al. 2020), cancers are the first or second leading cause of death in 112 of 183 countries worldwide, posing a severe threat to human health, and overall the global burden of cancer morbidity and mortality will continue to increase (Wen et al. 2021). Currently, surgery is still the primary method to treat solid cancers, combined with radiotherapy and chemotherapy, including a variety of cytotoxic drugs, tyrosine kinase inhibitors, in addition to immunotherapy such as immune checkpoint inhibitors, such as anti-programmed cell death 1 (PD-1), anti-programmed cell death ligand 1 (PD-L1), and anti-cytotoxic T lymphocyte-associated protein 4 (CTLA-4) antibodies (Xu et al. 2018). However, these approaches often fail to achieve satisfactory clinical results in cancer treatment (Vasan et al. 2019).

Gas signaling molecules are small molecule gases that affect cellular biology by regulating signal transduction, such as nitric oxide (NO) (Tien Vo et al. 2021), carbon monoxide (CO) (Oliveira et al. 2018), and hydrogen sulfide (H_2_S) (Flannigan and Wallace 2015). Studies have confirmed that multiple gas signaling molecules have anti-tumor properties and can be used directly or as products of specific agents for anti-tumor treatment.

Recently, studies have proved H_2_ to be another gas signaling molecule showing intriguing potential in cancer therapy (Wu et al. 2019; Li et al. 2019). Since 1975, when Dole et al. (1975) found that high concentrations of H_2_ could cure squamous cell carcinoma implanted in the skin of mice, numerous laboratory and clinical studies have confirmed that H_2_ is effective against various cancers (Wu et al. 2019; Li et al. 2019; Hirano et al. 2021a). Furthermore, H_2_ effectively synergizes with anti-tumor therapies such as radiotherapy and cytotoxic drugs (Runtuwene et al. 2015; Meng et al. 2015; Hirano et al. 2021b), reducing damage to body (Yang et al. 2012) and improving patient prognosis.

In a landmark study in 2007 (Ohsawa et al. 2007), Oshawa et al. found that H_2_ could selectively neutralize highly toxic reactive oxygen species (ROS) (hydroxyl radicals, ·OH, and peroxynitrite, ONOO–) without affecting other physiological ROS. The ROS-scavenging capacity of H_2_ is likely to be a critical underlying mechanism for its anti-tumor activity. However, the underlying mechanism of hydrogen in tumor therapy is controversial due to the lack of specific signaling receptors that other gas signaling molecules have. In this review, we first discuss the importance of H_2_ metabolism by the intestinal flora under physiological conditions for the homeostasis of the human internal environment. Then we discuss the mechanism of H_2_ anti-tumor through its unique antioxidant capacity to provide a comprehensive account of the mechanism of hydrogen action in tumor therapy. At last, we discussed the specific role of different application modalities of H_2_ and explored the prospect of hydrogen application in clinical tumor therapy.

## Anti-tumor barrier: H_2_ produced by intestinal flora

Under normal physiological conditions, adult gut microbiota can produce large amounts of H_2_ daily (Mego et al. 2017; Carbonero et al. 2012), and this H_2_ can regulate the balance of intestinal flora and their metabolites, which are essential for immune homeostasis in humans. It demonstrates that H_2_ is the body’s natural anti-tumor barrier and provides new strategies for its clinical use.

### H_2_ metabolism in  intestine

The intestinal hydrogenogenic bacteria mainly use various indigestible carbohydrates as substrates for anaerobic oxidative energy production, including starch, cellulose, and some sugars (Jiang et al. 2020). This process can produce large amounts of H_2,_ which is quickly absorbed and used by hydrogenotrophic bacteria. H_2_ participates in this series of reactions as an electron transporter and is a vital energy substance for the survival and proliferation of intestinal flora (Carbonero et al. 2012; Greening et al. 2016). Most of the H_2_ not used by the flora is excreted through respiration and the anus, while the rest can enter the circulation or penetrate the intestinal lumen and peritoneum into the peritoneal cavity (Nishimura et al. 2013).

The hydrogenotrophic bacteria mainly include reductive acetate-producing bacteria, sulfate-reducing bacteria (SRB), and methanogenic bacteria, which, respectively, oxidize H_2_ to acetate, H_2_S, and CH_4_ (Carbonero et al. 2012). The H_2_ concentration in the intestine not only passively responds to the balance of these florae but also controls the balance of hydrogenogenic and hydrogenotrophic flora by partial pressure of hydrogen (pH_2_) (Carbonero et al. 2012). For example, a study found (Ge et al. 2022) that hydrogen-rich water (HRW) supplementation significantly inhibited the expansion of opportunistic pathogenic *E. coli* and increased intestinal integrity in mice with colitis by modulating intestinal flora H_2_ metabolism.

Studies have confirmed that intestinal flora disorders can affect the occurrence and development of cancers in multiple organs throughout the body, including colorectal cancers (Song et al. 2020; Helmink et al. 2019). Although hydrogenogenic and hydrogenotrophic microbes cover most intestinal flora, the specific morphology and metabolism of the flora contained in them vary greatly and lack proper taxonomy, so there are few articles directly studying the relationship between intestinal H_2_ metabolizing and cancers. Several studies investigated the genomic and meta-genomic distribution of hydrogenases, the reversible enzymes that catalyze the oxidation and evolution of H_2_, to understand more about the contribution of H_2_ metabolism to gut ecosystems (Greening et al. 2016; Peters et al. 2015; Suzuki et al. 2018). According to the binding metal cofactor, Greening et al. identified 4 groups (22 subgroups) of [NiFe]-hydrogenases, 3 groups (6 isoforms) of [FeFe]-hydrogenases, and a small group of [Fe]-hydrogenases (Greening et al. 2016). This hydrogenase diversity supports crucial metabolic pathways of intestinal flora, such as H_2_-based respiration, fermentation, and carbon fixation processes, reflecting the scope of H_2_ metabolism in sustaining the growth and survival of microorganisms. Until now, the authors indicated that most related studies focus on only a few branches of the hydrogenase phylogenetic tree and a small fraction of organisms within the universal tree of microorganisms.

However, it is easy to find that H_2_ produced by intestinal flora can participate in the regulation of various flora metabolites related to carcinogenesis (Ge et al. 2022; Fan and Pedersen 2021; Kalantar-Zadeh et al. 2019), such as H_2_ can scavenge ROS and promote the production of short-chain fatty acids (SCFAs). For example, a study demonstrated that oral administration of HRW in mice could promote the production of SCFAs in cecal contents and circulation by modulating the composition of intestinal flora (Higashimura et al. 2018).

**Fig. 1:**
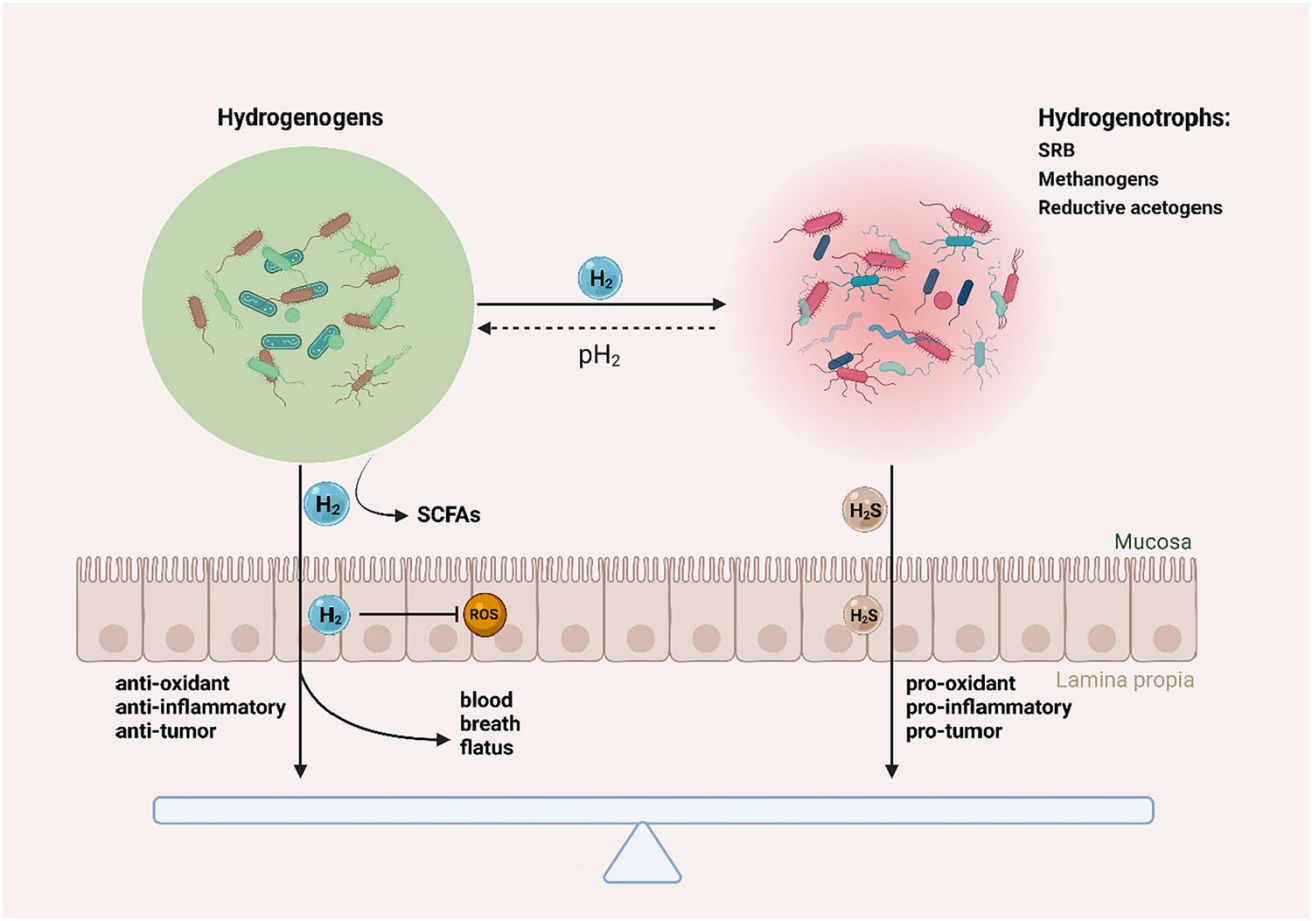
H_2_ metabolism in intestinal flora regulates intestinal health. Hydrogenogens produce H_2_ during fermentation in the human colon and result in a rapid increase of pH_2_, which restrict further fermentation. Three groups of hydrogenotrophic (H_2_ utilizing) microbes can oxidize H_2_, simultaneously lowering pH_2_ and enabling fermentation to continue. H_2_S produced by SRB can damage the intestinal epithelium and induce inflammation and tumorigenesis, while H_2_ can antagonize the malignant effects of H_2_S by producing SCFA and scavenging ROS. SCFAs: short-chain fatty acids; SRB: sulfate-reducing bacteria; pH_2_: partial pressure of hydrogen

In contrast, high SRB and sulfur protein diets are associated with the development of colon cancer (Nguyen et al. 2020; Lee et al. 2022), which can disrupt cytochrome oxidase, inhibit butyrate utilization, block mucus synthesis, and cause DNA methylation through the production of H_2_S. In addition, excessive H_2_S production by intestinal flora plays an important role in the carcinogenesis and development of intestinal tumors (Ngowi et al. 2021; Attene-Ramos et al. 2010; Dalal et al. 2021). Paradoxically, various sulfur-containing diets have long-proven anti-tumor properties, such as garlic and cruciferous vegetables (Rose et al. 2021; Abbaoui et al. 2018). We think part of the reason is the balance of SRB with other hydrogen-metabolizing flora in the lumen in those different dietary settings. Moreover, the balance of H_2_ and H_2_S metabolized by SRB may be the underlying mechanism (Fig. 1).

###  H_2_ produced by intestinal flora can maitain homeostasis

**Fig. 2:**
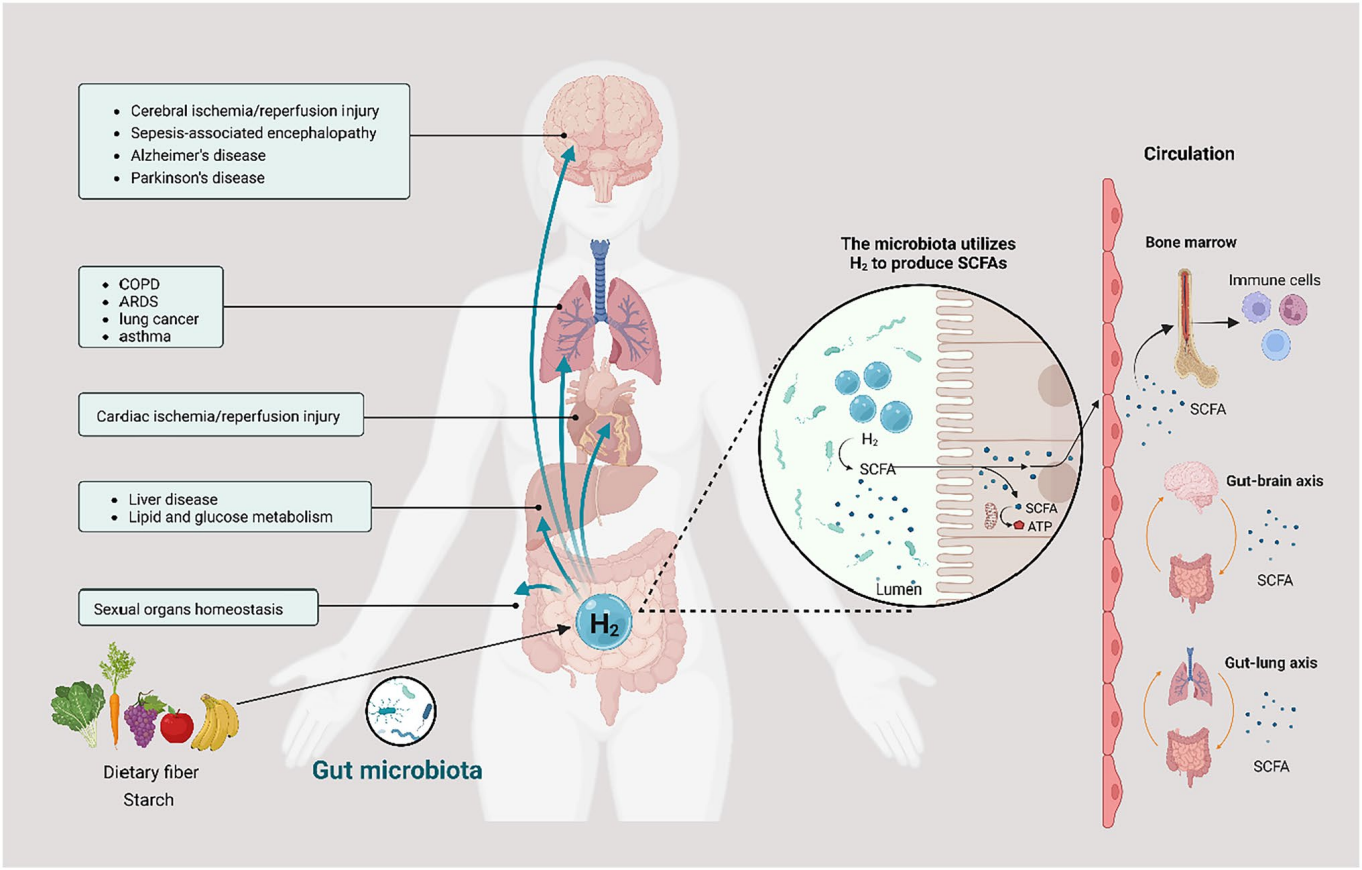
H_2_ produced by intestinal flora maintains multi-system health and immune homeostasis. The hydrogen produced in the intestine can penetrate the abdominal cavity or bloodstream, exerting a protective effect on multiple organs throughout the body. On the other hand, hydrogen is a substrate for SCFA production by intestinal flora. It promotes the production of SCFA, a vital energy substance for intestinal epithelial cells and immune cells, maintaining the integrity of the intestinal barrier and the stability of the systemic immune system. COPD: chronic obstructive pulmonary disease; ARDS: acute respiratory distress syndrome; SCFA: short-chain fatty acid

Intestinal H_2_ metabolizing is not only fundamental to gut health but also crucial for redox balance and immune homeostasis in multiple organs (Fig. 2).

Studies have shown that H_2_ maintains the integrity of the intestinal barrier, reduces intestinal inflammation and damage in rat (Wu et al. 2017), and protects ischemia–reperfusion of the brain, lung, liver, and other organs (Dong et al. 2018; Fang et al. 2018; Sano et al. 2018; Ishikawa et al. 2018). H_2_ is also fundamental to pelvic health and guarantees organ function (Zhang et al. 2021). In male and female organs, such as testes and ovaries, after damage leading to dysfunction, H_2_ supplementation can effectively reduce oxidative stress and promote recovery of organ function. Although many studies have focused on exogenous H_2_ supplementation, H_2_ production in the gut is as effective as H_2_ inhalation and H_2_ oral HRW in raising H_2_ levels in the body. For example, the administration of fructose promotes an 11-fold increase in intraperitoneal H_2_ concentration and a significant increase in H_2_ in the blood system (Nishimura et al. 2013).

SCFA is an essential source of energy for intestinal epithelium and multiple immune cells (Yip et al. 2021), as well as a communication material between the gut–lung axis and gut–brain axis (Fulling et al. 2019) that sustains immune homeostasis throughout the body. H_2_, produced by gut bacteria, is the substrate for SCFAs synthesis and promotes the synthesis of SCFAs. It was found that HRW can increase propionate, butyric acid, and total SCFAs in the gut by regulating gut flora to treat brain diseases such as Parkinsonism (Bordoni et al. 2019; Ostojic 2021). Diets or medications that promote H_2_ production in the gut, such as high-fiber diets and lactose, also promote the production of SCFAs (Chen and Vitetta 2018; Zhai et al. 2018). A study in mice (Ge et al. 2022) suggested that HRW can strengthen the intestinal barrier by regulating specific mucositis-related mucolytic bacteria through the H_2_–H_2_ metabolic microbiota–SCFAs axis, ensuring the body’s internal environment.

## Anti-tumor effect of H_2_ and the underlying mechanism

### H_2_ anti-tumor and synergistic anti-tumor effects

Starting with the research by Dole et al. (1975) that high concentrations of H_2_ cured squamous carcinomas grown on mouse skin, several studies have confirmed the anti-tumor effects of H_2_. Wang et al. (2018) reported that in cells and mice, H_2_ inhibited the proliferation, metastasis, and invasion of lung cancer cells and reduced lung cancer volume by inhibiting chromosome stabilization protein 3 (SMC3). A clinical study by Akagi and Baba (2019) found that inhalation of H_2_ for 3 h daily significantly prolonged progression-free survival and overall survival in stage IV colon and rectal patients. A study of inhaled H_2_ in 82 cases of intermediate to advanced cancer treatment also confirmed the anti-tumor effect of H_2_ (Chen et al. 2020).

Chemotherapy and radiotherapy are still the main strategies for cancer treatment. However, these treatment regimens lead to significant oxidative stress and inflammation, causing damage to human organs, and H_2_ can be used as an adjuvant regimen to suppress these adverse effects due to its cytoprotective properties, such as antioxidant and anti-inflammatory (Meng et al. 2015). Runtuwene et al. (2015) gave HRW drinking to colorectal cancer-bearing mice treated with 5-fluorouracil intravenously. They found that HRW enhanced apoptosis of cancer cells by causing significant increases in the expression of p-AMPK, apoptosis-inducing factor (AIF), and caspase-3 in non-cancer cells and prolonging the life span of cancer-bearing animals. Cisplatin causes the accumulation of ROS in humans, decreases glutathione activity, and causes increased oxidative stress, while H_2_ reverses cisplatin-induced oxidative stress in the body and restores antioxidant enzyme activity (Kikkawa et al. 2014). In addition, H_2_ reduces cisplatin’s nephrotoxicity without affecting its anti-tumor effect and enhances animal survival in mice experiments. Oral administration of HRW (0.55–0.65 mM, 1.5–2.0 L per day) to patients with hepatocellular carcinoma receiving radiotherapy suppressed the level of oxidative stress in patients and improved their quality of life without affecting the effect of radiotherapy (Nakashima-Kamimura et al. 2009). Some studies reported that the administration of inhaled H_2_ during radiotherapy treatment reduced the damage to the hematological and immune systems (Hirano et al. 2021b; Yang et al. 2012) and alleviated the growth of radiotherapy-induced thymic lymphoma (Zhao et al. 2011).

Although a series of studies have confirmed the anti-tumor effects of H_2_, it is indispensable to understand the underlying mechanisms in depth to support further H_2_ application in clinic. H_2_ has an extensive range of physiological effects, including anti-oxidative stress, anti-inflammation, and regulation of apoptosis (Li et al. 2019). Furthermore, some studies proved that H_2_ has an anti-tumor effect by indirectly regulating gene expression (Hirano et al. 2021a). Through these studies, we believe that the ability of H_2_ to selectively scavenge highly toxic ROS may be the core and fundamental mechanism of its anti-tumor effects, so this paper mainly focuses on this point of discussion.

### H_2_ anti-tumor activity through anti-oxidative stress

Intracellular ROS are mainly derived from catalytic reactions regulated by oxidative phosphorylation (OXPHOS) of the mitochondrial respiratory chain and NADPH oxidase (NOX) in the cytoplasm (Holmstrom and Finkel 2014). On the one hand, ROS are extremely oxidative and destructive to biomolecules proteins, phospholipids, and nucleic acids; on the other hand, ROS are key intracellular signaling molecules that can affect cell proliferation and differentiation by regulating various signaling pathways, such as NF-κB and Akt/mTOR (Cheung and Vousden 2022; Zhang et al. 2016). Under normal physiological conditions, the complete system of antioxidant enzymes in the body keeps the ROS concentration in a precise dynamic balance, including superoxide dismutase (SOD), which converts ${\text{O}}_2^{\cdot -}$ to H_2_O_2_, then glutathione peroxidase (GPx) and catalase (CAT) convert H_2_O_2_ to water (Cheung and Vousden 2022; Meng et al. 2021). However, the body lacks specific scavenging systems for ·OH and ONOO–, and these two ROS are highly cytotoxic and have damaging effects on almost all macromolecules (proteins, nucleic acids, lipids), which can lead to DNA double-strand structure disruption and base pairing damage (Cheung and Vousden 2022; Jena 2012), resulting in carcinogenesis.

Activation of oncogenes altered mitochondrial function (Ismail et al. 2019), and hypoxia collectively contribute to increased ROS production in cancer cells. Unfortunately, the antioxidant enzyme system in tumor cells is often unable to counteract overgenerated ROS, resulting in a high ROS state in the tumor microenvironment (Cheung and Vousden 2022; Zhang et al. 2016; Liao et al. 2019; Hornsveld and Dansen 2016). Indeed, cancer cells can not only adapt to a moderately high ROS state but also take advantage of ROS to drive the malignant phenotype. This happens because ROS can enhance NF-κB, Akt/mTOR, Wnt/β-catenin pathways, and oncogenes such as Ras, Bcr/Abl, and c-Myc expression (Cheung and Vousden 2022; Liao et al. 2019; Hornsveld and Dansen 2016; Wojtovich et al. 2019), which maintain high-intensity metabolism and proliferation of tumor cells. Furthermore, ROS-dependent signaling pathways can promote cancer invasion and metastasis (Cheung and Vousden 2022; Liao et al. 2019). However, some reports (Cheung and Vousden 2022; Hornsveld and Dansen 2016) suggest that persistently elevated ROS in the cancer microenvironment can limit further cancer progression after reaching a certain level. Thus, non-selective antioxidant therapy in cancer treatment may lead to further cancer progression (Meng et al. 2021; Sayin et al. 2014; Chandel and Tuveson 2014). H_2_ selectively removes strongly oxidizing without affecting other ROS, making it an ideal therapy antioxidant.

On the one hand, H_2_ can inhibit the damage of cellular DNA by ·OH and ONOO– to prevent cancer development; on the other hand, H_2_ can remove ROS from cancer cells and inhibit multiple ROS-dependent metabolic signaling pathways to suppress cancer development. Studies have confirmed that H_2_ can effectively reduce oxidative stress caused by various pathological conditions, including cancers, and promote the restoration of redox homeostasis (Adzavon et al. 2022; Shi et al. 2016; Kawai et al. 2012).

H_2_ can also elevate the expression of some antioxidant enzymes that play a crucial role in regulating redox homeostasis in cancer cells (Li et al. 2019; Slezak et al. 2021), which exerts anti-tumor effects. Some non-cancer studies proved that H_2_ treatment induced a significant increase in the expression of intracellular SOD, GPx, CAT (Zhou et al. 2019), and heme oxygenase-1 (HO-1) (Fang et al. 2018; Iketani et al. 2017), enhancing their potential to eliminate ROS.

**Fig. 3:**
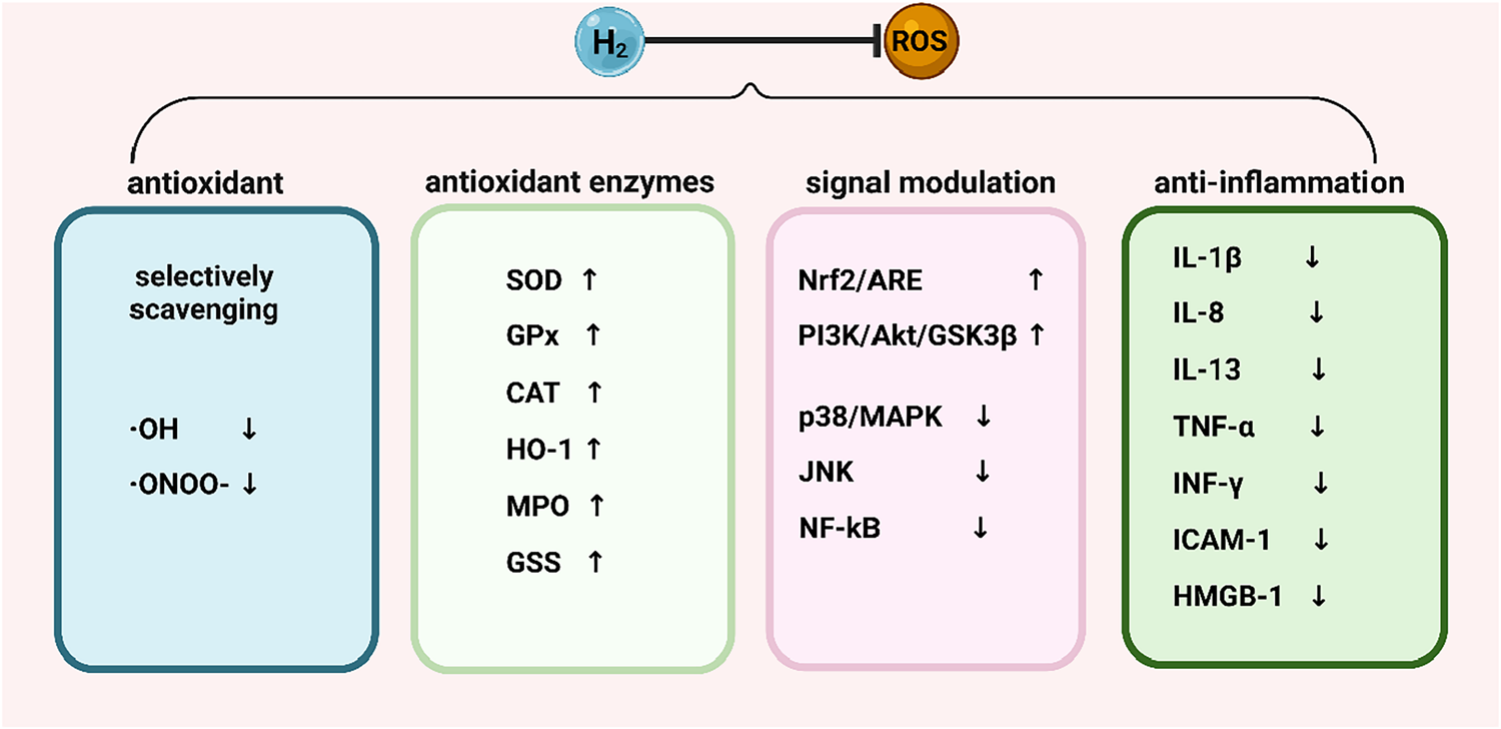
Mechanisms of H_2_ in anti-oxidative stress. SOD: superoxide dismutase; CAT: catalase; GPx: glutathione peroxidase; HO-1: heme oxygenase 1; MPO: myeloperoxidase; GSS: glutathione synthetase; MAPK: mitogen-activated protein kinase; JNK: c-Jun N-terminal kinase; Nrf2: nuclear factor-erythroid-2-related factor 2; ARE: antioxidant response elements; NF-κB: nuclear factor kappa B; TNF-α: tumor necrosis factor-alpha; ICAM-1: intercellular cell adhesion molecule-1; IFN-γ: interferon-gamma; IL-1β: interleukin beta; IL-8: interleukin 8; IL-13: interleukin 13; HMGB-1: high-mobility group box protein 1

H_2_’s ability to modulate various signaling pathways is another essential mechanism for its antioxidant action, such as Nrf2/ARE and p38/MAPK (Fang et al. 2018; Slezak et al. 2021; Xie et al. 2020). A series of subsequent studies have found that H_2_ also maintains redox balance in the body by activating the Keap1-Nrf2-ARE, and Nrf2-HO-1 pathways (Slezak et al. 2021; Xie et al. 2020; Yu et al. 2019; Chen et al. 2015), which exerts immunomodulatory, anti-inflammatory, and cancer pro-apoptotic effects. Wang et al. (2018) found that H_2_ inhibited ROS expression and increased SOD, IL-1β, IL-8, IL-13, and tumor necrosis factor-α (TNF-α) expression in lung tissue of cancer-bearing mice. (Fig. 3).

### The immunoprotective function of H_2_

Many clinical trials have confirmed the role of H_2_ in modulating cancer immunity. A 2018 clinical study involving 55 stage IV colon cancer patients showed that inhalation of H_2_ reduced PD-1 expression on CD8^+^ T cells in the patient’s peripheral blood, reduced CD8^+^ T cell depletion, and improved prognosis (Akagi and Baba 2019). In a clinically advanced small cell lung cancer study, continuous H_2_ inhalation for 2 weeks reversed the suppressed intrinsic and adaptive immune systems in patients’ peripheral blood, reduced depleted CD8^+^ T cells, and restored functional CD4^+^, CD8^+^ T cells, and natural killer cell ratios to normal levels (Chen et al. 2020). Although few studies investigate the underlying mechanisms, the ability to selectively clear toxic ROS and protect T cell mitochondria may be the core mechanism of H_2_’s immune protection function.

**Fig. 4:**
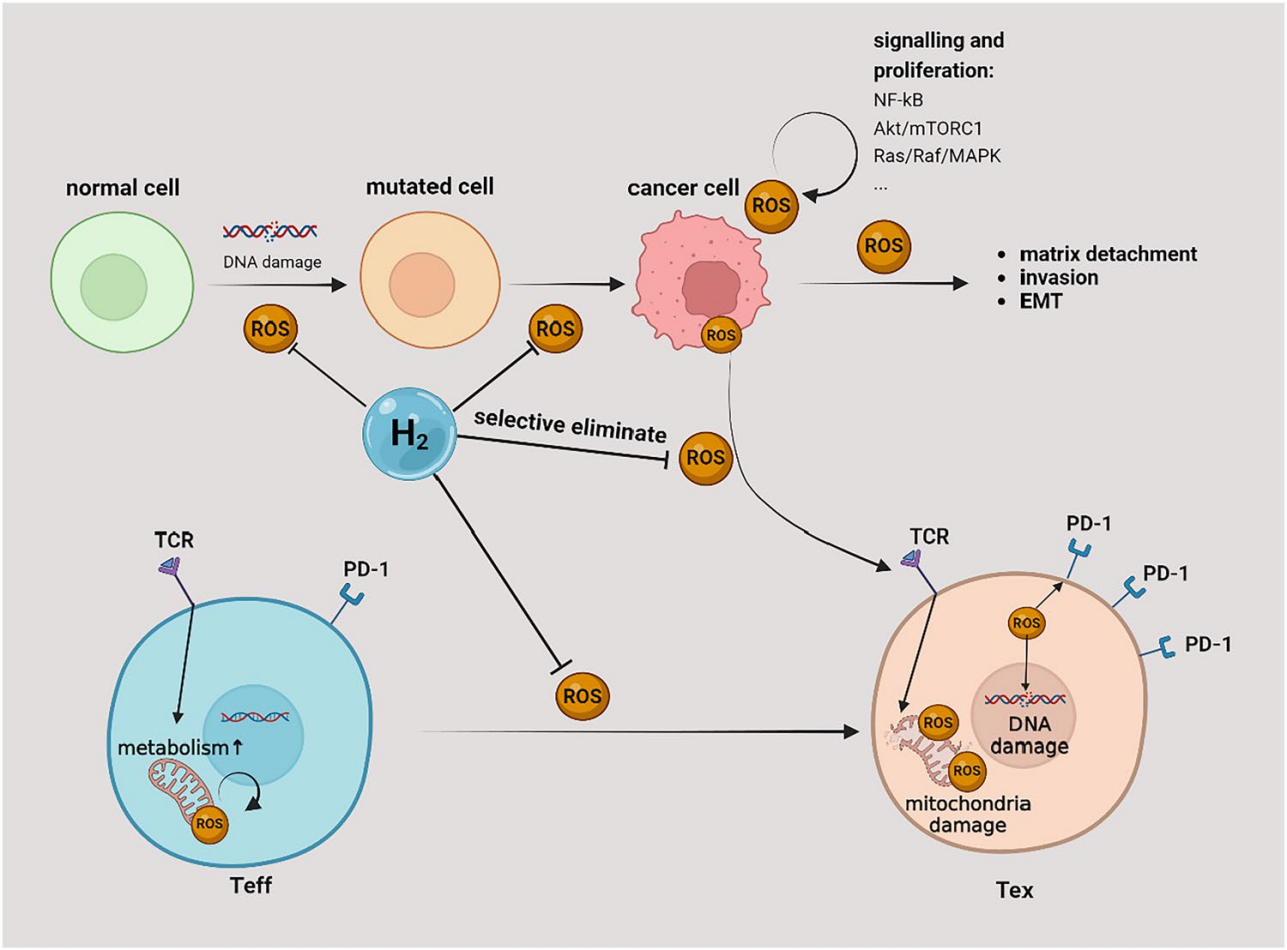
H_2_ anti-tumor activity by selectively eliminating ROS in the tumor microenvironment. ROS can cause tumorigenesis by damaging DNA, leading to genetic mutations, and regulating various crucial signaling pathways leading to tumor development and metastasis. ROS in the tumor microenvironment can infiltrate T cells, increasing the oxygen pressure burden of continuously activated T cells. Excessive ROS in T cells can interfere with mitochondrial energy metabolism, damage T cell DNA, and promote PD-1 expression, leading to T cell dysfunction and apoptosis. ROS: reactive oxygen species; EMT: epithelial–mesenchymal transition; Teff: effector T cells; Tex: exhausted T cells. NF-κB: nuclear factor kappa B; MAPK: mitogen-activated protein kinase; mTORC1: mammalian target of rapamycin complex 1

After T cell receptor (TCR) activation by antigen-presenting cancer antigens, downstream signal transduction enhances mitochondrial metabolism, and ROS, the apparent byproducts of mitochondrial metabolism, are significant molecules that regulate multiple core pathways involved in T cell metabolic recombination (Franchina et al. 2018; Franco et al. 2020). However, as previously noted, overgrowing cancer cells can cause elevated ROS in the cancer microenvironment, and mitochondria produce high ROS when T cells are activated, in addition to increased ROS in T cells due to factors such as hypoxia (Scharping et al. 2021), which results in tumor-infiltrating lymphocytes (TILs) facing a far higher physiological state of ROS when activated (Franco et al. 2020). Sustained high ROS levels disrupt T cell mitochondria, inhibit T cell activation and lead to T cell dysfunction by deflecting T cell metabolic restructuring (Laura 2012; Scharping et al. 2016), and promote PD-1 expression to induce apoptosis (Najjar et al. 2019) (Fig. 4). H_2_, on the other hand, combats oxidative stress in various disease conditions and restores redox balance in the body’s environment by regulating the NADH/NADPH pathway (Adzavon et al. 2022; Tao et al. 2019), thereby safeguarding T cell activation and preventing apoptosis. Moreover, in this condition, compared with routine anti-tumor medicine, the high permeability of H_2_ grants it to easily penetrate inside the tumor, even into structures such as the mitochondria of the TILs.

Since the mitochondrial respiratory chain is the primary source of ROS production in cells, high local concentrations of ROS can lead to mutations of mitochondrial DNA and direct disruption of mitochondrial dynamics, ultimately leading to mitochondrial metabolic dysfunction (Deng et al. 2020) and apoptosis of T cells(Scharping et al. 2021). The study shows that TILs in renal cell carcinoma contain hyperpolarized, fragmented mitochondria producing significant ROS (Siska et al. 2017). Akagi et al. found in clinical studies of lung cancer patients (Akagi and Baba 2019, 2020) that inhalation of H_2_ enhances the mitochondrial function of CD8^+^ T cells and decreases PD-1 expression in the patient’s peripheral blood, suggesting that H_2_ may act by regulating peroxisome proliferators activate receptor-γ coactivator-1α (PGC-1α). Mo et al. (2019) suggested that in vitro H_2_ can enter mitochondria to neutralize toxic ROS, attenuate mitochondrial oxidative stress damage, protect Na^+^/Ka^+^ ATP pumps, enhance Bcl-2 expression, inhibit voltage-dependent anion channel 1 (VDAC1) expression and opening, protect mitochondrial membranes, and also inhibit the release of apoptotic factors such as caspase 9.

## Application of H_2_

### Exogenous H_2_ administration

**Fig. 5:**
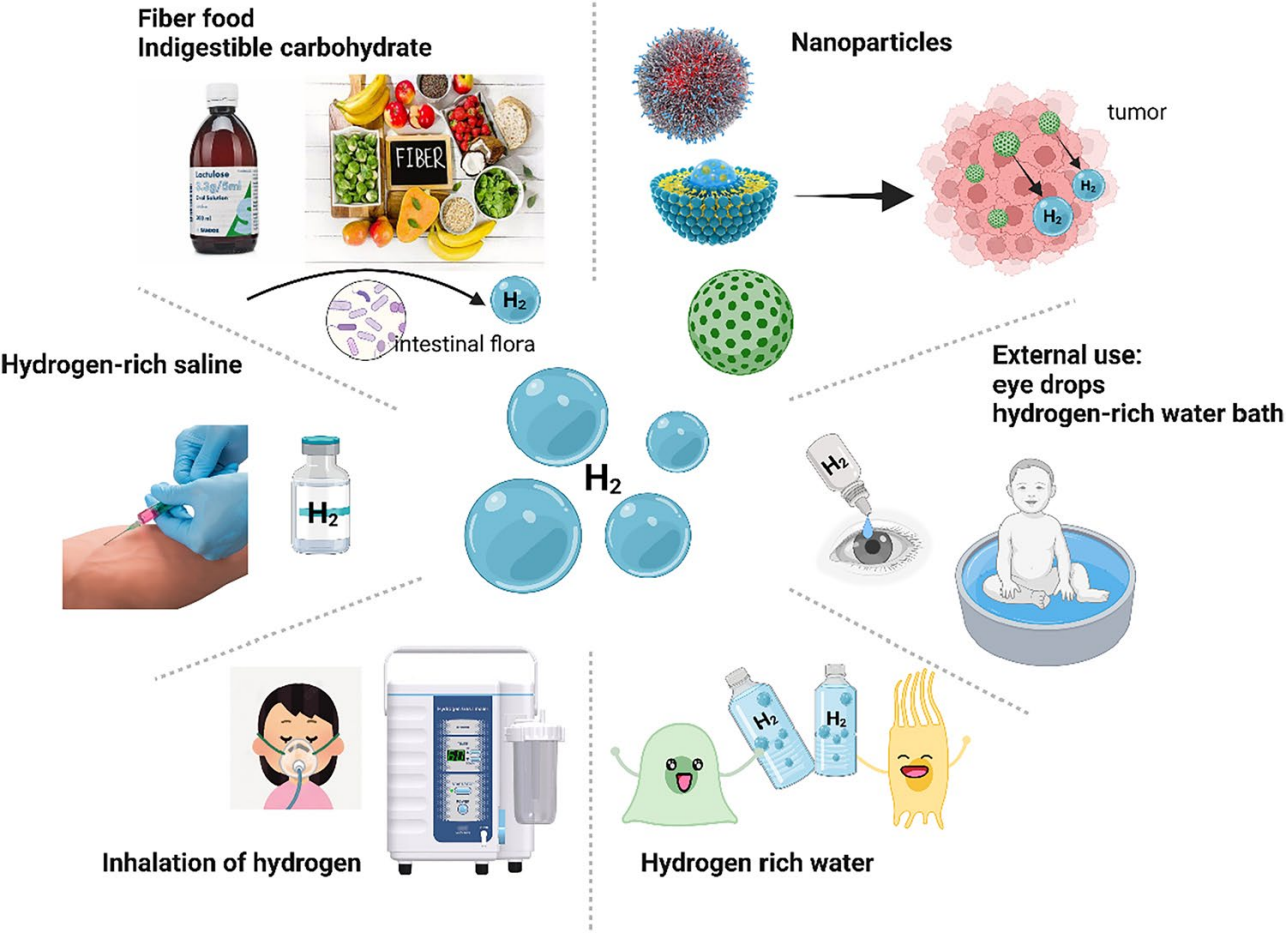
Various applications of H_2_

The conventional ways of exogenous administration of H_2_ are inhalation, oral HRW, injection of saline containing H_2_, and external use, such as eye drops and HRW baths (Fig. 5). Some reviews have compared the rates of H_2_ concentration increase in different body organs caused by different H_2_ application methods and their therapeutic effects (Li et al. 2019; Hirano et al. 2021a), and we will not present them here individually. However, we cannot simply correlate the role of different H_2_ application methods in disease treatment with H_2_ concentrations in the circulatory and respiratory systems—for example, the unique role of HRW in regulating intestinal flora and energy metabolism.

Some studies suggest that HRW can regulate intestinal flora and contribute to restoring and maintaining the intestinal flora’s homeostasis (Higashimura et al. 2018; Kajiyama et al. 2008). In 2018, Japanese scholars (Higashimura et al. 2018) found that oral administration of HRW for 4 weeks improves the distribution of flora in the colon, increases SCFA production, and decreases plasma cholesterol concentration. Xiao et al. (2018) found that HRW could affect intestinal flora by regulating the expression of MyD88, thereby reducing the injury from abdominal radiotherapy and increasing survival and body weight after radiotherapy in mice. HRW has also interacted with diet to enhance and prolong hepatic H_2_ accumulation (Kamimura et al. 2011), lower blood lipids and glucose, and promote the direct secretion of brain intestinal peptides from intestinal epithelial cells (McCarty 2015). In one article (Ito et al. 2012), HRW prevented the development of 6-hydroxydopamine-induced Parkinson’s disease in mice, whereas continuous H_2_ inhalation and oral lactulose were less effective. Although the article did not explore the underlying mechanisms of this phenomenon, the unique physiological effects of HRW, such as the regulation of intestinal flora, may contribute to it.

Some scientists have designed nanoparticles that can release large amounts of H_2_ at cancer sites (Wu et al. 2019, 2021; Sun et al. 2020), providing the possibility of precise local production of sustained high concentrations of H_2_ to enhance the anti-tumor effect, and the combined application of nanotechnology and H_2_ may be an important direction for future precision cancer therapy. For example, Zhang et al. constructed covalently loaded liposomes with semiconductor polymers-Pdots as catalysts (Zhang et al. 2019), a “nanoscale H_2_ factor” containing reactants, intermediates, and byproducts, which can continuously produce H_2_ at the site by laser stimulation and effectively reduce cancer growth in mice. Sun et al. designed a laser-triggered H_2_ release nanoparticle to enhance the chemotherapeutic effect of mouse bladder cancer and reduce the toxic response of chemotherapeutic drugs (Sun et al. 2020). Wu et al. constructed Au-TiO2@ZnS nanoparticles that can release H_2_ triggered by in vitro X-ray under the guidance of in vitro photoacoustic imaging, achieving an excellent therapeutic effect and mild inflammatory response in combination with radiotherapy for in situ liver cancer in mice (Wu et al. 2021).

### Regulating intestinal flora production of H_2_

In addition to the exogenous H_2_ supplementation mentioned above, supplementation with high fiber, indigestible starches, and sugars can also increase intestinal H_2_ production through intestinal flora, which is the most suitable and economical treatment for daily life (Fig. 5).

Lactulose is a disaccharide that cannot be absorbed by the body and can promote large amounts of H_2_ production by intestinal flora, thus effectively increasing the concentration of H_2_ in the human abdominal cavity and blood. Studies have confirmed that lactulose can relieve inflammation and injury in multiple organs such as the intestine and brain by promoting intestinal H_2_ production, such as mitigating ulcerative colitis caused by the carcinogenic substance dextran sodium sulfate (DSS) (Zhai et al. 2013; Chen et al. 2013). Perlamutrov et al. (2016) found that lactulose can treat dermatitis by stimulating H_2_ and SCFA production. Studies have confirmed that oral administration of lactulose or dietary fiber containing indigestible starch and dietary fiber can regulate intestinal flora (Jiang et al. 2020; Trompette et al. 2014), balance the intestinal environment, and have therapeutic effects on multi-system diseases such as chronic obstructive pulmonary disease (COPD) and neurological disorders (Vaughan et al. 2019; Kong et al. 2021). Although some researchers have attributed much of the clinical effects of lactulose and fibrates to specific intestinal flora and SCFAs, H_2_ may be an overlooked link in these experiments (Kalantar-Zadeh et al. 2019; Desai et al. 2016). Similar drugs, fructans, and inulin, also have anti-inflammatory and metabolic-modulating effects by promoting intestinal H_2_ production (Nishimura et al. 2013).

Perioperative dietary management has profound meaning for the long-term prognosis of cancer patients. To ensure the energy requirements and enhance the immunity of cancer patients, some researchers have proposed the concept of an immunonutrition diet (Adiamah et al. 2021; Svetikiene et al. 2021; Prieto et al. 2017), including glutamine, arginine, sulfur-containing amino acids, and polyunsaturated fatty acids. However, such a high-protein, high-fat diet may cause elevated blood glucose and lipids and metabolic disorders in patients on the one hand, and poor dietary choice may cause adverse emotions in patients on the other hand. As mentioned earlier, oral HRW has a good energy regulation function, which can improve the liver energy metabolism of the body, lower blood lipid glucose, and reduce the side effects of a high-fat diet (Qiu et al. 2020). In contrast, some studies show that fiber and indigestible starch diets have anti-inflammatory and anti-tumor effects (Jiang et al. 2020; Trompette et al. 2014; Desai et al. 2016). Therefore, a fiber-rich diet or oral HRW combined with an immunonutritional diet may be a more suitable dietary strategy for cancer patients in the perioperative period.

### H_2_ in perioperative applications

**Fig. 6:**
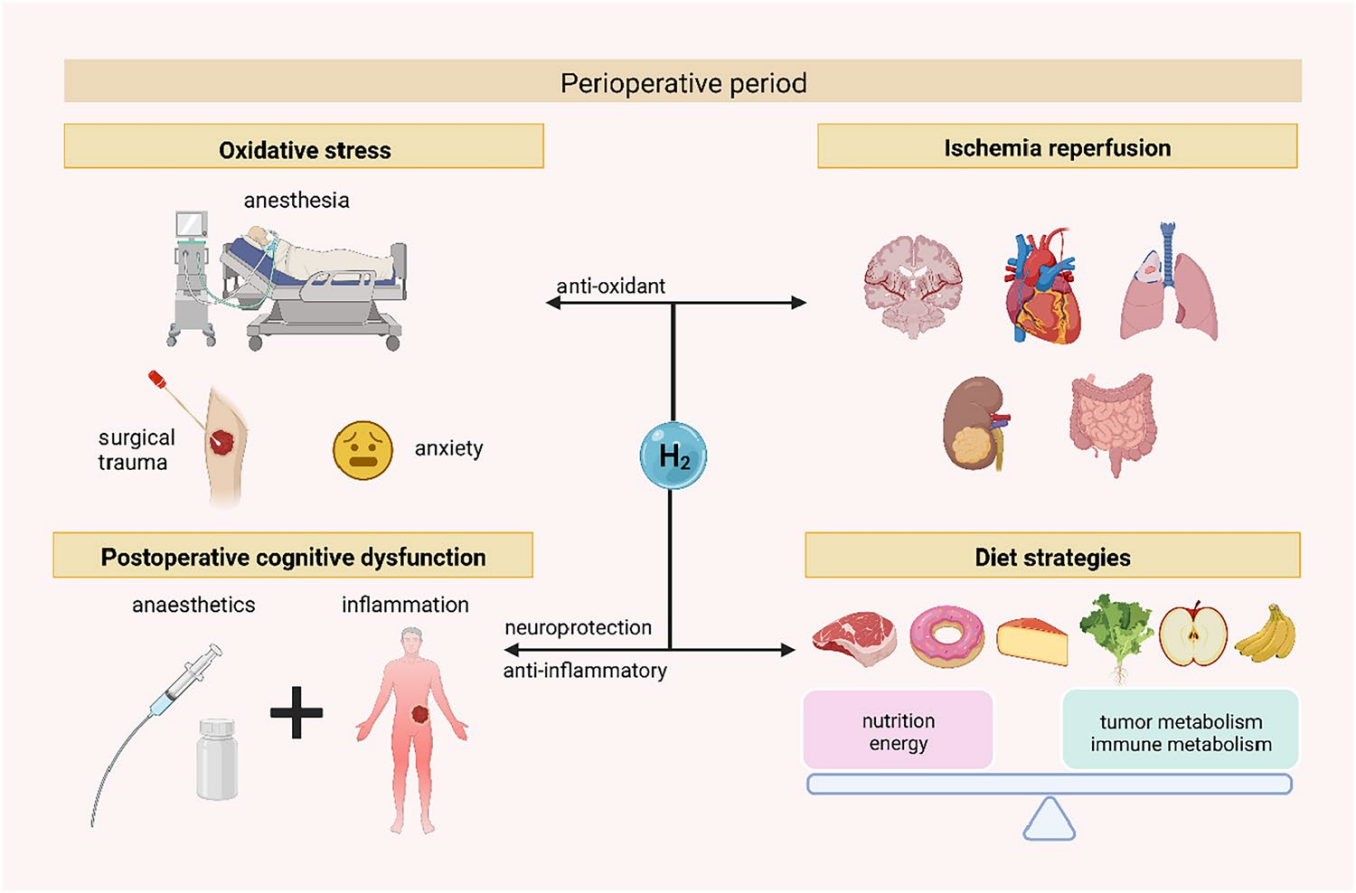
The multiple roles of hydrogen in the perioperative period for oncology patients. Hydrogen has been reported to have antioxidant and anti-inflammatory effects and to improve ischemia–reperfusion in the heart, brain, lungs, and other organs, making it possible to alleviate perioperative oxidative stress and ischemia–reperfusion injury in vital organs. Hydrogen also has a neuroprotective function, antagonizing the damage to the central nervous system caused by anesthetics and systemic inflammation. In addition, hydrogen-rich water has energy-regulating and intestinal flora-modulating effects, which are also valuable in the perioperative dietary management of oncology patients

Clinically, tumors often need surgical treatment, so they inevitably face multiple perioperative stress factors such as trauma, anesthesia, and mental stress. Rapidly elevated oxidative stress can lead to an imbalance of internal environmental homeostasis and immune system suppression in tumor patients while promoting tumor recurrence and metastasis (Hsiao et al. 2021; Matzner et al. 2020; Seckler et al. 2020). H_2_ has physiological effects of antioxidant, anti-inflammatory, and immune modulation, which can effectively antagonize these unfavorable factors (Sano et al. 2018) (Fig. 6).

In addition, H_2_ can effectively alleviate ischemia–reperfusion injury in multiple organs (Dong et al. 2018; Xie et al. 2020; Fu and Zhang 2022). For example, in a randomized controlled clinical trial of 26 patients (Ono et al. 2017), Ono et al. found that 3% H_2_ inhaled twice daily for 1 h significantly improved vital signs, stroke scale scores, physiotherapy index, and 2-week brain MRI in stroke patients compared with conventional treatment.

Postoperative cognitive dysfunction (POCD) is a postoperative complication in patients undergoing clinical procedures and is particularly prevalent in older patients. Currently, the recognized etiology of POCD is neuroinflammation caused by the combined effects of anesthetics and surgery-induced systemic inflammation (Lai et al. 2021). H_2_ supplementation alleviates symptoms of central nervous system disorders such as Parkinson’s disease and autism by redressing intestinal flora imbalance (Suzuki et al. 2018; Kong et al. 2021; Doifode et al. 2021). Li et al. (2010) reported that intraperitoneal injection of hydrogen-rich saline effectively alleviated central nervous system inflammation and oxidative stress and reduced cognitive impairment in mice. Therefore, some researchers have stated that H_2_ can be used for neuroprotection in perioperative patients (Wang et al. 2020) (Fig. 6).

## Conclusion and perspectives

The H_2_ produced by intestinal flora is a natural antioxidant in the body’s internal environment, which can regulate oxidative stress caused by various reasons in the body’s internal environment and become a natural barrier against carcinogenesis and development. H_2_ is also a substrate for the production of SCFAs through the intestinal flora, essential substances for maintaining the stability of the body’s immune system and affecting the gut–brain axis and the gut–lung axis. However, there may be more potential mechanisms for why H_2_ has such a wide range of effects. For example, HRW can promote the production of ghrelin (McCarty 2015), which is crucial in regulating food intake and energy homeostasis. Therefore, whether H_2_ can affect the body’s immune system by regulating the secretion of other flora metabolites or affecting energy metabolism by other gastrointestinal hormones needs further investigation.

Intestinal flora and cancers have long attracted widespread medical attention, and scholars in several countries have actively studied the relationship between genomics and metabolomics of intestinal flora and cancers (Song et al. 2020; Fulling et al. 2019; Erny and Prinz 2020). In those studies, researchers suggest that sulfate-reducing bacteria are related to colorectal cancer (Nguyen et al. 2020). More analysis of a large sample of intestinal flora genomics from the perspective of H_2_ metabolizing flora and insight into the association between intestinal flora H_2_ metabolism and cancer may be able to find new drug targets and guide the subsequent application of H_2_ in cancer therapy. For example, some researchers explored the significance of the H_2_ metabolism of microbiota through genomic and meta-genomic surveys of the distribution of hydrogenase subtypes (Greening et al. 2016; Peters et al. 2015).

Studies have shown that H_2_ inhalation for about 2 weeks can restore the body’s redox balance and enhance immune cells’ function in the peripheral blood of cancer patients, which indicates that H_2_ has a protective effect on the overall immune system of the body. However, in-depth studies on the specific effects of H_2_ on TILs in the cancer microenvironment and its potential mechanisms are still lacking. However, according to some studies (Akagi and Baba 2020), H_2_ can protect the mitochondria of TILs by scavenging ROS, preventing the differentiation of TIL toward the end-stage phenotype, and acting as a qualified adjuvant immunotherapeutic agent. Therefore, its synergistic therapeutic effects with immune checkpoint blockers are yet to be studied.

Furthermore, some studies suggested HRW can regulate the energy metabolism of hepatocytes and adipocytes (Kajiyama et al. 2008; Kamimura et al. 2011; Acker et al. 2021; Iio et al. 2013). Although it remains to be investigated whether H_2_ can play a role in energy metabolism in cancer cells or immune cells similar to that in hepatocytes and adipocytes, those researches indicated that HRW may exert a more comprehensive potential in anti-tumor immunotherapy by regulating immunometabolism.

There is often cross-talk between gas signaling molecules. For example, H_2_S and NO can regulate each other’s production and enhance each other’s anti-tumor effects (Jing et al. 2021). Moreover, it is not difficult to find the intrinsic connection between H_2_ and other gas signaling molecules, such as sulfate-reducing bacteria can metabolize H_2_ to produce H_2_S, which are in some delicate balance in the intestine. H_2_ can regulate CO production through HO-1 (Yu et al. 2019), and H_2_ can inhibit inducible nitric oxide synthase (iNOS) and enhance the expression of endothelial nitric oxide synthase (eNOS) (Slezak et al. 2021). Combining H_2_ with other gas signaling molecules may be the development direction of H_2_ for cancer treatment, and some studies have suggested this idea in nanotechnology (Jing et al. 2021).

## Data Availability

No datasets were generated or analysed during the current study.

## Abbreviations

AIF: Apoptosis-inducing factor
AMPK: Adenosine monophosphate activated protein kinase
ARE: Antioxidant response element
CAT: Catalase
CH_4_: Methane
CO: Nitric oxide
COPD: Chronic obstructive pulmonary disease
CTLA4: Cytotoxic T lymphocyte-associated protein 4
DNA: Deoxyribonucleic acid
DSS: Dextran sodium sulfate
FGF21: Fibroblast growth factor
GHS-R1a: Secretion stimulating receptor for endogenous growth hormone-1a
GPx: Glutathione peroxidase
GSM: Gas signaling molecules
H_2_: Hydrogen
H_2_O_2_: Hydrogen peroxide
H_2_S: Hydrogen sulfide
HO-1: Heme oxygenase-1
HRW: Hydrogen rich water
IL: Interleukin
iNOS: Inducible nitric oxide synthase
Keap1: Kelch-like ECH-associated protein 1
mTOR: Mammalian target of rapamycin
NADH: Nicotinamide adenine dinucleotide
NADPH: Nicotinamide adenine dinucleotide phosphate
NF-kb: Nuclear transcription factor kappa b
NO: Nitric oxide
NOX: NADPH oxidase
Nrf2: Nuclear factor erythroid-2-related factor 2
OXPHOS: Oxidative phosphorylation
PD-1: Programmed cell death 1
PD-L1: Programmed cell death ligand 1
PGC-1α: Peroxisome proliferators activate receptor-γ coactivator -1α
pH_2_: Partial pressure of hydrogen
POCD: Postoperative cognitive dysfunction
ROS: Reactive oxygen species
SCFA: Short-chain fatty acid
SMC3: Chromosome stabilization protein 3
SOD: Superoxide dismutase
SRB: Sulfate-reducing bacteria
TCR: T cell receptor
TILs: Tumor-infiltrating T cells
TNF-α: Tumor necrosis factor-α
VDAC1: Voltage-dependent anion channel 1
WHO: World Health Organization
